# Early access to biological neonatal screening: coordination among child care action programs

**DOI:** 10.1590/1518-8345.2938.3266

**Published:** 2020-05-11

**Authors:** Beatriz Molina Carvalho, Waldomiro Roberto Tavares, Jéssica Batistela Vicente, Gabriel Zanin Sanguino, Adriana Moraes Leite, Maria Cândida de Carvalho Furtado

**Affiliations:** 1 Universidade de São Paulo, Escola de Enfermagem de Ribeirão Preto, PAHO/WHO Collaborating Centre for Nursing Research Development, Ribeirão Preto, SP, Brazil.; 2 Universidade Estadual de Londrina, Hospital Universitário, Londrina, PR, Brazil.; 3 Centro Universitário Barão de Mauá, Ribeirão Preto, SP, Brazil.; 4 Secretaria Municipal de Saúde, Programa de Atenção Integral à Saúde da Criança e do Adolescente, Ribeirão Preto, SP, Brazil.; 5 Scholarship holder at the Conselho Nacional de Desenvolvimento Científico e Tecnológico (CNPq), Brazil.; 6 Universidade Estadual de Maringá, Maringá, PR, Brazil.

**Keywords:** Neonatal Screening, Health Care Surveys, Health Services Coverage, Child Care, Newborn, Delivery of Health Care, Triagem Neonatal, Pesquisas sobre Serviços de Saúde, Cobertura de Serviços de Saúde, Cuidado da Criança, Recém-nascido, Assistência à Saúde, Tamizaje Neonatal, Encuestas de Atención de la Salud, Cobertura de los Servicios de Salud, Cuidado del Niño, Recién Nacido, Prestación de Atención de Salud

## Abstract

**Objective:**

To verify factors associated with early newborn access to biological neonatal screening.

**Method:**

A cross-sectional quantitative study was carried out with all newborns who underwent tests in healthcare units, hospitals, and laboratories of a city in the state of São Paulo, Brazil, with programs linking healthcare information. The following variables were investigated: child’s age at collection (dependent); place of collection; date of collection; and type of user (independent). Descriptive and inferential statistics were applied.

**Results:**

Records of 15,652 screenings were found in the two years analyzed. In the first year analyzed, 7,955 births and 7,640 (96.0%) tests were recorded, of which 5,586 (73.1%) were undertaken with newborns between three and five days old. In the next year analyzed, 8,316 births and 8,012 (96.3%) screenings were recorded, of which 7,025 (87.6%) were undertaken with newborns in the same age group. A statistically significant association was found between the variables “child’s age” and “type of user” in one year, and between the variables “child’s age” and “place of collection” in both years.

**Conclusion:**

Early access to these tests enables the screening of diseases and referral for treatment. The present study contributes to the management of child care programs by presenting strategies linking data and actions to improve access to biological neonatal screening.

## Resumo

**Objetivo:**

verificar fatores associados ao acesso precoce de recém-nascidos à triagem neonatal biológica.

**Método:**

estudo transversal, quantitativo, com todos os bebês que realizaram exame em unidades de saúde, hospitais ou laboratórios de um município do estado de São Paulo, Brasil, com programas que vinculam informações de atenção à saúde. Foram investigadas as variáveis: idade da criança na coleta (dependente), local da coleta, data da coleta, tipo de usuário (independentes). Aplicou-se estatística descritiva e inferencial.

**Resultados:**

há registro de 15.652 triagens nos dois anos analisados. No primeiro ano analisado, ocorreram 7.955 nascimentos e 7.640 (96,0%) exames, 5.586 (73,1%) deles em recém-nascidos de três a cinco dias de vida. Para 8.316 bebês nascidos no ano seguinte, foram registradas 8.012 (96,3%) triagens, 7.025 (87,6%) delas na mesma faixa etária. Encontrou-se associação estatisticamente significativa entre as variáveis “idade da criança” e “tipo de usuário” em um ano, e entre “idade da criança” e “local da coleta” em ambos os anos.

**Conclusão:**

o acesso precoce ao exame oportuniza a triagem de doenças e o encaminhamento para tratamento. O estudo contribui com a gestão de programas de atenção à criança, ao apresentar estratégias que articulam informações e ações para melhoria do acesso à triagem neonatal biológica.

**Objetivo:**

verificar factores asociados al acceso precoz del recién nacido al tamizaje neonatal biológico.

**Método:**

estudio transversal, cuantitativo, con todos los bebés examinados en unidades de salud, hospitales o laboratorios de un municipio del estado de São Paulo, Brasil; con programas integradores de información de atención de salud. Fueron investigadas las variables: edad del niño en la recolección (dependiente), lugar de recolección, fecha de recolección y tipo de usuario (dependientes). Se aplicó estadística descriptiva e inferencial.

**Resultados:**

existen registrados 15.652 triajes en los dos años estudiados. Durante el primero, ocurrieron 7.955 nacimientos y 7.640 (96,0%) análisis, 5.586 (73,1%) en recién nacidos de tres a cinco días de vida. Hubo 8.316 nacimientos en el año siguiente, se registraron 8.012 triajes, 7.025 (87,6%) para la misma faja etaria. Se encontró asociación estadísticamente significante entre las variables “edad del niño” y “tipo de usuario” en un año, y entre “edad del niño” y lugar de recolección” en ambos años.

**Conclusión:**

el acceso precoz al análisis permite el tamizaje de enfermedades y la derivación para tratamiento. El estudio contribuye a la gestión de programas de atención infantil, presentando estrategias que articulan información y acciones para mejorar el acceso al tamizaje neonatal biológico.

## Introduction

The objective of comprehensive child health care is to promote quality of life and reduce child mortality. As a way to operationalize this care, the National Policy for Comprehensive Child Health Care (PNAISC, its acronym in Portuguese), in its strategic areas, directs and organizes this care by means of definition of priority actions for child health care, such as addressing perinatal issues, which are the main causes of infant mortality^(^ 1 ^)^.

Prior to the PNAISC, more than a decade ago, the Commitment Agenda for Comprehensive Child Health Care and Infant Mortality Reduction introduced comprehensive care lines in order to provide care continuity, with proposals on actions ranging from health promotion and disease prevention to treatment and rehabilitation. The third care line of this document, “neonatal screening: neonatal heel prick,” focuses on an important test carried out with newborns that prioritizes early detention of possible diseases, as well as subsequent treatment and appropriate follow-up^(^ 2 ^-^ 5 ^)^.

The National Neonatal Screening Program, established in 2001, integrates biological, hearing, and eye neonatal screening^(^ 1 ^)^. The program is committed to developing policies, programs (child health care and care for people with disability), and care networks of the Brazilian Unified Health System (SUS, its acronym in Portuguese), such as the Stork Network and the Care Network for the Disabled Person^(^ 1 ^)^.

Biological neonatal screening, which is the theme of the present study, has as its purpose, by means of blood collection in the heel area of newborns (NB), early detection of metabolic, genetic, enzymatic, and endocrine disorders, such as: phenylketonuria; congenital hypothyroidism; sickle cell anemia and other hemoglobinopathies; cystic fibrosis; biotinidase deficiency; and congenital adrenal hyperplasia. Although they are commonly asymptomatic in the neonatal period, these conditions have a high potential for causing damage, with repercussions on children’s growth and development^(^ 1 ^,^ 6 ^)^.

Infants who present results indicating any type of disorder must be monitored and receive medical follow-up during treatment^(^ 3 ^-^ 5 ^)^. For appropriate identification of diseases, newborns should have their blood collected between the third and fifth day of life, and women should receive guidance, while still in pregnancy, on the need for and importance of this test, and become aware that families have the right of access to its results^(^ 1 ^,^ 7 ^-^ 8 ^)^.

Studies carried out in Brazil have shown evolution in the coverage of neonatal screening in the North^(^ 9 ^)^, Northeast^(^ 10 ^)^, and South^(^ 11 ^)^ regions, with the last showing optimization of arrival time of samples for analysis^(^ 11 ^)^, which enables early diagnosis and calling families in for the beginning of appropriate treatment of infants in cases of positive results^(^ 1 ^,^ 11 ^)^.

Biological neonatal screening is extremely important for children’s health, and they have the right to care, access to early diagnosis, and referral to specialized care services within an appropriate time^(^ 2 ^-^ 5 ^,^ 7 ^)^.

The commitment of Brazilian cities to the organization and implementation of the National Neonatal Screening Program was essential for these results, as well as the active participation of municipal health departments (SMS, its acronym in Portuguese), through control actions and monitoring of healthcare units^(^ 1 ^)^. These measures effectively contributed to achieving the program’s objectives^(^ 7 ^,^ 10 ^)^.

In the city where the present study was carried out, located in the state of São Paulo, Brazil, the Municipal Neonatal Screening Program has, as coordinator of its actions, the program *Floresce uma Vida* (A Life Blooms), which is linked to the Comprehensive Child and Adolescent Care Program of the Municipal Health Department. One of the actions of this program is scheduling the collection of material necessary for biological neonatal screening in primary health care network units, in the case of children born in public maternity hospitals in the city. This action of offering infants pre-scheduled appointments in healthcare units aims to ensure their attendance in the period recommended for the test. This coordinated care action is relevant to meet the recommendations of public care policies for this population.

Therefore, considering the importance of providing infants with safe access and timeliness of health care, meeting a public care agenda for this population^(^ 1 ^,^ 6 ^-^ 7 ^)^, the objective of the present study was to verify factors associated with access of NBs to biological neonatal screening in 2016 and 2017 in a city in the state of São Paulo, by means of coordination of two healthcare programs.

## Method

This was a cross-sectional study with a quantitative approach, carried out in a medium-sized city in the state of São Paulo, Brazil, which has five health regions, called “health districts” (central, southern, eastern, northern, and western). Primary healthcare units and family healthcare units in all these regions carry out blood collection for biological neonatal screening. Regarding the hospital network, the city has eight hospitals, of which five provide care for users of the SUS (four provide childbirth and birth care) and three are private hospitals. In addition, the city has eight private laboratories that also undertake screening tests.

To operationalize the flow of NB after discharge from maternity hospitals that provide care for users of the SUS to healthcare units, as well as to ensure that biological neonatal screening is carried out within the recommended time frame, while the NB are still in the hospital maternity units, a nursing team of the program *Floresce uma Vida* schedules dates for the tests, on the same day as the first appointment with the nurse in the healthcare unit, between three and five days of life^(^ 12 ^)^.

Since the program *Floresce uma Vida* acts in public hospital maternities in the city, this type of service is not available for children born in private hospitals; however, some of these NB get to primary healthcare units by spontaneous demand. In addition, some infants born in these hospitals have their blood collected for biological neonatal screening in their own hospitals or, by parental choice, in private laboratories in the city. Every month, all locations that collect blood for the test in the city fill in a form and send it to the nurse who is responsible for the Municipal Neonatal Screening Program, who records the data in a single worksheet for follow-up and evaluation.

All NB residents in the city who underwent biological neonatal screening from January 1, 2016 to December 31, 2017, in primary healthcare units, hospitals, or private laboratories, were included in the study. The data, extracted from worksheets of the Municipal Neonatal Screening Program in February 2018, contained the following information: place and date of blood collection; hospital name; date of birth; and type of user. This last item was subdivided into public healthcare services, defined as SUS users, and private healthcare services, indicated as SUS non-users (those who make use of public healthcare services only for biological neonatal screening collection). The city also meets childbirth and delivery demands from other locations; however, it does not undertake biological neonatal screening for these infants. Therefore, infants who did not reside in the city were excluded.

The following variables were analyzed: “place of collection”, a categorical variable made up of healthcare unit, hospital, or laboratory; “child’s age at collection,” a continuous variable obtained by calculating the difference between the date of collection and the date of birth; and “type of user,” a categorical variable made up of SUS users and SUS non-users. It is worth mentioning that the age variable was categorically described in the database, with the following four intervals: three to five days; six to seven days; eight to ten days; and >11 days.

Since the database was digitized in Excel worksheets, the processing was carried out by means of management of this database. The Statistical Package for the Social Sciences 16.0 (SPSS) was used for this purpose.

In the analysis, the NB were characterized regarding the variables of interest, seeking for information identifying access to biological neonatal screening within the period recommended by the Brazilian Ministry of Health. The period from three to five days of life was defined as early access^(^ 7 ^)^. In addition, possible associations between the dependent variable “child’s age at collection” and the independent variables “place of collection” (healthcare unit, hospital, and laboratory) and “type of user” (SUS users and SUS non-users) were investigated, by means of application of the chi-squared test.

The present study met the ethical precepts of Resolution 466/2012 of the Brazilian National Health Council, and the project was approved by the Research Ethics Committee of University of São Paulo at Ribeirão Preto College of Nursing, under protocol no. 2.490.469 and Certificate of Presentation for Ethical Consideration CAAE no. 64258717.7.0000.5393.

Informed consent forms were not required, because data collection was carried out only through worksheet consultation and did not include face-to-face participation of the NB. The database was handled with complete confidentiality, respecting the anonymity of the infants and healthcare institutions.

## Results

In the two years analyzed in the present study, 15,652 screenings were undertaken. In 2016, 7,955 births were recorded and 7,640 (96.0%) tests were undertaken, of which 5,586 (73.1%) were for NB between three and five days of life. For 8,316 infants born in 2017, 8,012 (96.3%) screenings were recorded, with 7,025 (87.6%) for the same age group.

For most of the infants who underwent biological neonatal screening, their material was collected in healthcare units (Table 1).

**Table 1:** Univariate analysis of the association between year and place of collection by the biological neonatal screening, 2016-2017. Ribeirão Preto, São Paulo, Brazil, 2018

Variables	Hospitals/Laboratories		Healthcare units		p-value*
	N	%	N	%	
2016					0.0000^†^
Three to five days	2136	38.2	3450	61.7	
2016					0.0000^†^
Six to seven days	301	19.0	1280	81.0	
>7 days	114	24.1	359	75.9	
2017					0.0000^†^
Three to five days	2804	39.9	4221	60.1	
Six to seven days	208	26.6	573	73.4	
>7 days	68	33.0	138	67.0	

* p < 0.05;

† Cochran-Mantel-Haenszel test

Source: Municipal Neonatal Screening Program. Ribeirão Preto, São Paulo, Brazil

Of the total tests carried out in 2016 up to the seventh day of life, early access was identified for 87.6% of the infants in hospitals and laboratories, and 72.9% in healthcare units. In the following year, there was a 5% increase in collections in hospitals and laboratories, which represented 93.1% of tests in the first week of life. In healthcare units, there was a 15% increase compared with the previous year, accounting for 88.0% of the tests undertaken.

Regarding the analysis of healthcare units, most collections were for SUS users, in both years evaluated (Table 2).

**Table 2:** Univariate analysis of the association between the year of biological neonatal screening collection and type of user in healthcare units, 2016-2017. Ribeirão Preto, São Paulo, Brazil, 2018

Variables	SUS*		SUS^†^		p-value^‡^
	N	%	N	%	
2016					0.0000^§^
Three to five days	2418	70.1	1032	29.9	
Six to seven days	1033	80.7	247	19.3	
>7 days	269	74.9	90	25.1	
2017					0.517^§^
Three to five days	3316	78.6	905	21.4	
Six to seven days	452	78.9	121	21.1	
>7 days	114	82.6	24	17.4	

* Users of the Unified Health System;

† Non-users of the Unified Health System;

‡ p < 0.05;

§ Cochran-Mantel-Haenszel test

Source: Municipal Neonatal Screening Program. Ribeirão Preto, São Paulo, Brazil

In the two years analyzed, a higher frequency of early test collection was found for all infants who underwent the test in healthcare units, for both SUS users and SUS non-users.

To verify associations between the study’s variables of interest, values of the intervals “eight to ten days” and “>11 days” were grouped; these were considered beyond the period recommended for carrying out this test. The Cochran-Mantel-Haenszel test found a statistically significant association between “child’s age” and “place of collection” for both years, as indicated in Table 1. The variable “type of user” was considered when collections were only analyzed in healthcare units, with a statistically significant association for 2016, as shown in Table 2.

## Discussion

With the purpose of providing appropriate treatment to NB in the pre-symptomatic phase for possible congenital diseases^(^ 2 ^-^ 4 ^)^, biological neonatal screening must be undertaken between the third and fifth day of life, in order to provide early diagnosis of these diseases and initiate appropriate care^(^ 3 ^,^ 5 ^,^ 7 ^)^. The results of the present study show that the city is meeting this recommendation, since the mean of collection by healthcare units in the period recommended as ideal was considered higher, compared to tests undertaken after the fifth day of life.

Evaluations of coverage in other Brazilian regions have also presented significant results, evidencing the effectiveness of a program directed to this type of test, which, when well-based and organized, is able to provide appropriate care in newborns’ first week of life^(^ 9 ^)^.

The commitment of the city to the management of the neonatal screening program significantly contributes to an increase in records for NB, as well as collection and sending of the test to the reference service in neonatal screening^(^ 10 ^)^.

Regarding the present study, biological neonatal screening tests were carried out for more than 90% of the NB, most in healthcare network units of the city. These values were higher than those found by other authors (77.1%)^(^ 13 ^)^. In 2016 and 2017, 93% and 97% of the newborns, respectively, underwent collection in the first week of life, and these rates were also higher than those found in the previously mentioned study, which indicated 87.7%^(^ 13 ^)^. The data showed that private and public services in the city met the recommendation of the Ministry of Health regarding the ideal period for test collection^(^ 6 ^)^ for most of the NB studied.

An increase in early access to this test was found from 2016 to 2017, with associations between “child’s age” and “place of collection” for both years, and “type of user” for 2016. Studies have highlighted the importance of early diagnosis of the diseases investigated in biological neonatal screenings, with the purpose of providing appropriate treatment in order to reduce negative repercussions on children’s health^(^ 2 ^-^ 5 ^,^ 14 ^-^ 16 ^)^.

Two of the variables investigated, “place of collection” and “type of user,” indicated the influence of the program *Floresce uma Vida* in the search for access by NB to biological neonatal screening. In addition to other care actions previously mentioned, this program allows NB to receive care in their first week of life in healthcare network units of the city, and this has contributed to the comprehensive health of this population^(^ 12 ^,^ 17 ^-^ 18 ^)^. Therefore, the joint efforts of the two programs, *Floresce uma Vida* and the Municipal Neonatal Screening Program, allow NB early access to healthcare actions.

Coordination of biological neonatal screening flows, with integration among primary health care, specialized care, and hospital maternities, allows the organization of healthcare levels^(^ 1 ^)^. This integration, in turn, favors comprehensive care and organized access in units of healthcare services^(^ 1 ^,^ 12 ^)^, as evidenced in actions developed among the abovementioned programs.

A study carried out with nurses of the Brazilian Health Family Strategy in this same city indicated the importance, for these professionals, of the actions developed by the program *Floresce uma Vida* regarding the early access of NB to healthcare services, especially for care actions such as biological neonatal screening^(^ 18 ^)^.

In addition to organizational efforts by the municipal health department of each city to establish and meet the goals of the National Neonatal Screening Program, according to the needs of each region, knowledge developed and shared between healthcare professionals and mothers/families of NB is essential to appropriately carrying out the test. However, one study showed that mothers’ knowledge about this test did not cover its entire meaning, but was restricted to specific guidance provided by healthcare professionals who instructed them to take the NB to a healthcare unit within the ideal period^(^ 19 ^)^. It is worth mentioning that mothers often receive this recommendation at a moment of fragility during hospital discharge, simultaneously with a lot of other information (health surveillance with follow-up of child growth and development, immunization, birth certificate, and breastfeeding). This may lead to lack of understanding regarding the diseases investigated by this test and the need to undertake it between the newborn’s third and fifth day of life.

In this context, nurses play an important role in teaching about and carrying out the neonatal screening process, since they are the professionals who are most in contact with mothers and NB, in addition to other family members. Therefore, they can make use of valuable tools such as education and health promotion, and take advantage of situations such as prenatal care, to build, together with mothers, knowledge regarding the test. It is worth mentioning that, according to the needs of each patient, these meetings may last longer, and, when they are well-conducted and concluded, the women are better prepared, biologically and psychologically, to absorb the concepts learned^(^ 19 ^-^ 20 ^)^.

The reasons that some infants underwent biological neonatal screening at a higher age than that recommended were not identified, which is considered a limitation of the present study. This may have occurred due to lack of understanding by those responsible for newborns regarding guidance on this test, both in the prenatal period and hospital maternities. Another hypothesis would be the operationalization of the program *Floresce uma Vida* in situations such as weekends, holidays, or lack or absence of healthcare professionals, making it difficult for some SUS users to schedule the biological neonatal screening at the appropriate time. However, because this was not the focus of the present study, further studies could indicate the reasons that some collection occurred after the recommended time, once the city introduced, more than two decades ago, scheduling of this test while newborns were still in the maternity hospitals of the primary health care system.

In terms of a contribution to and advancement of scientific knowledge, the present study allows managers of child healthcare programs, at the local, regional, and national levels, to observe positive aspects for improving care for this population. The program *Floresce uma Vida* is an example. It enables the scheduling of several care actions in healthcare units, including the biological neonatal screening test, and provides mothers (SUS users) who are still in the maternity hospital with documents showing the date when they must take their children to the healthcare service. In addition, it is worth mentioning the partnership of this program with the Municipal Neonatal Screening Program, linking important information for the management of child care.

## Conclusion

The present study emphasized that infants born and residing in the city investigated in 2016 and 2017 had early access to biological neonatal screening. Both primary healthcare units and private hospitals and laboratories satisfactorily met the official recommendations of the Brazilian Ministry of Health. Collecting material for the test in healthcare units and being SUS users was statistically significant, which may have been mostly favored by coordination among the city’s programs.

In conclusion, while an action aims to identify alterations in the health of infants to refer them, in due time, to appropriate care, the management of the Municipal Neonatal Screening Program has successfully achieved a significant number of newborns who underwent collection for the test before completing the first week of life.

This program also depends on the aid of the program *Floresce uma Vida*, which schedules the test and provides guidance to puerperal women (SUS users) who are still in the maternity hospital regarding the importance of infant care in the first days of life (first consultation in the primary health care, carrying out biological neonatal screening, and immunization). These actions have enabled the arrival of infants in healthcare units right after hospital discharge, with access to this service and care continuity initiated in maternity hospitals. However, for tests collected beyond the recommended time, further studies must seek the reasons for their occurrence.

Infants born in the city rely on the management of two programs that link care actions to enable carrying out biological neonatal screening while infants are still in the first week of life. Initiatives of this nature increase the chance of early diagnosis for appropriate treatment, minimize diseases in the health of infants, and continuously contribute to qualifying the care provided to this population.
